# Phytochemical Analysis, Antioxidant, Antimicrobial, and Cytotoxic Activity of Different Extracts of *Xanthoparmelia stenophylla* Lichen from Stara Planina, Serbia

**DOI:** 10.3390/plants11131624

**Published:** 2022-06-21

**Authors:** Aleksandar Kocovic, Jovana Jeremic, Jovana Bradic, Miroslav Sovrlic, Jovica Tomovic, Perica Vasiljevic, Marijana Andjic, Nevena Draginic, Mirjana Grujovic, Katarina Mladenovic, Dejan Baskic, Suzana Popovic, Sanja Matic, Vladimir Zivkovic, Nevena Jeremic, Vladimir Jakovljevic, Nedeljko Manojlovic

**Affiliations:** 1 Department of Pharmacy, Faculty of Medical Sciences, University of Kragujevac, 34000 Kragujevac, Serbia; salekkg91@gmail.com (A.K.); jovanabradickg@gmail.com (J.B.); sofke-ph@hotmail.com (M.S.); jovicatomovic2011@gmail.com (J.T.); andjicmarijana10@gmail.com (M.A.); nevenasdraginic@gmail.com (N.D.); sanjad.matic@gmail.com (S.M.); nbarudzic@hotmail.com (N.J.); mtnedeljko@gmail.com (N.M.); 2 Department of Biology and Ecology, Faculty of Sciences and Mathematics, University of Niš, 18000 Niš, Serbia; pericavasiljevic@gmail.com; 3 Department of Human Pathology, IM Sechenov First Moscow State Medical University (Sechenov University), 119991 Moscow, Russia; drvladakgbg@yahoo.com; 4 Department of Science, Institute for Information Technologies, University of Kragujevac, 34000 Kragujevac, Serbia; mirjana.grujovic@pmf.kg.ac.rs (M.G.); katarina.mladenovic@pmf.kg.ac.rs (K.M.); 5 Centre for Molecular Medicine and Stem Cell Research, Faculty of Medical Sciences, University of Kragujevac, 34000 Kragujevac, Serbia; dejan.baskic@gmail.com (D.B.); popovic007@yahoo.com (S.P.); 6 Institute of Public Health Kragujevac, 34000 Kragujevac, Serbia; 7 Department of Physiology, Faculty of Medical Sciences, University of Kragujevac, 34000 Kragujevac, Serbia; vladimirziv@gmail.com; 8 Faculty of Pharmacy, IM Sechenov First Moscow State Medical University (Sechenov University), 119991 Moscow, Russia

**Keywords:** ethnopharmacology, usnic acid, pharmaceutical potential, biological activities, flavonoid content, phenolic content, DPPH

## Abstract

The aim of this study was to identify some of the secondary metabolites present in acetonic, methanolic, and hexanic extracts of lichen *Xanthoparmelia stenophylla* and to examine their antioxidant, antimicrobial, and cytotoxic activity. Compounds of the depsid structure of lecanoric acid, obtusic acid, and atranorin as well as usnic acid with a dibenzofuran structure were identified in the extracts by HPLC. The acetone extract was shown to have the highest total phenolic (167.03 ± 1.12 mg GAE/g) and total flavonoid content (178.84 ± 0.93 mg QE/g) as well as the best antioxidant activity (DPPH IC_50_ = 81.22 ± 0.54). However, the antimicrobial and antibiofilm tests showed the best activity of hexanic extract, especially against strains of *B. cereus*, *B. subtilis,* and *S. aureus* (MIC < 0.08, and 0.3125 mg/mL, respectively). Additionally, by using the MTT method, the acetonic extract was reported to exhibit a strong cytotoxic effect on the HeLa and HCT-116 cell lines, especially after 72 h (IC_50_ = 21.17 ± 1.85 and IC_50_ = 21.48 ± 3.55, respectively). The promising antioxidant, antimicrobial, and cytotoxic effects of *Xanthoparmelia stenophylla* extracts shown in the current study should be further investigated in vivo and under clinical conditions.

## 1. Introduction

Despite the development of human medicine, cancers and infections caused by microorganisms and parasites continue to pose a serious threat to public health. In addition, inadequate use of antibiotics in recent decades has led to a significant increase in antibiotic resistance in some common pathogenic strains [1,2]. Given the proven health benefits of natural remedies so far, as well as the cultural shift from synthetic drugs to natural treatments for a wide range of diseases, it is not surprising that various natural compounds have been the focus of many studies in recent decades [3,4,5]. In that sense, even combinations of conventional medicines with natural products are being investigated in order to improve the effectiveness and reduce side effects [6].

In the quest for novel natural antimicrobial, anticancer, and antioxidant sources, our prime interest focused on lichens. As a community of two organisms—fungi (mycobiont) and algae (photobiont)—lichens grow on high mountains, pastures along with moss, eruptive rocks, soil, and tree bark as well as in forests and orchards and in tropical areas and on leaves. Since ancient times, lichens have had an important role in traditional medicine and the pharmaceutical industry as well as in human and animal nutrition [7,8]. Only in the last few decades has the number of studies focused on lichens with antimicrobial, anti-inflammatory, cytotoxic, antioxidant, antidiabetic, and analgesic activities grown enormously [9,10,11,12]. Additionally, formulations, such as liposomes and nanoparticles, are being developed with lichens or their metabolites as the main components [13,14,15]. Lichens represent a valuable source of diverse unique natural compounds. Namely, over a thousand different secondary metabolites from lichens have been isolated and identified until now, with a different chemical structure such as depsides, depsidones, dibenzofurans, and xanthones [7,15,16]. Furthermore, it is reported that lichen metabolites have biological significance and therapeutic value and can also be used as natural antioxidants or as an alternative to synthetic antifungal, antioxidative, and anticancer drugs [7,15].

*Xanthoparmelia stenophylla, Parmeliaceae* (synonyms *Xanthoparmelia somloensis*, *Parmelia stenophylla* (Ach.) Heugel, and *Parmelia conspersa* subsp. *Stenophylla* Ach.), is a cosmopolitan lichen that usually grows on a well-lit stone base. The talus is yellow–gray–green, up to 5 cm wide, firm in consistency, and loosely attached to the substrate on which it grows. The surface of lichens can be shiny with more or less pronounced white spots. The lichen lobes are up to 3 mm wide, elongated, dichotomously branched, pinnately divided, and overlapping. The lower surface is pale brown to brown, moderately rhizinic. Salazic and usnic acid have been identified as the main constituents of *X. stenophylla* lichen extracts so far [17]. *X. stenophylla* is traditionally used in the treatment of sexually transmitted diseases, such as syphilis, to reduce inflammation of the gingival tissue, and in the treatment after a snake bite. Moreover, other lichens from the genus *Xanthoparmelia* with traditional use have proven to be effective in the treatment of arthritis, rheumatism, chronic pain, swelling, increased menstrual bleeding, etc. [8].

Usnic acid is one of the most frequently tested secondary metabolites of lichens. It is most commonly found in lichens from the genera *Usnea* and *Xanthoparmelia* (family *Parmeliaceae*). This molecule has a dibenzofuranic structure and can naturally exist in two isomeric forms, (+) and (−) enantiomers, depending on the 9b positioned methyl group. Ever since it was first discovered and isolated in 1844, it has become worthy of research attention due to the fact of its various beneficial effects on human health. A variety of studies, both in vitro and in vivo, has confirmed antioxidant, antibacterial, antifungal, and antitumor effects of usnic acid per se [18,19,20]. Additionally, in addition to usnic acid, other lichen acids, including salazinic, obtusic, lecanoric acids, and atranorin, are highlighted as antioxidant components as well [10,18,19]. To the best of our knowledge, the pharmacological effects of *Xanthoparmelia stenophylla* have not been fully investigated so far; however, we may assume that this species extract will exert a significant therapeutic potential attributed to additive and/or synergistic effects of the aforementioned compounds.

The aim of this study was to identify some of the secondary metabolites present in acetonic, methanolic, and hexanic extract of the lichen *X. stenophylla*, as well as to examine the antioxidant, antimicrobial, and cytotoxic activity of these extracts. The special novelty of our research is reflected by revealing the influence of different extraction solvents on the biological activity of the previously mentioned extracts. Our findings could provide deeper insight into the potential health benefits of the lichen *X. stenophylla* and contribute to general knowledge about this lichen species.

## 2. Results

### 2.1. Yield

The extraction yields for *Xanthoparmelia stenophylla* acetonic extract (XSA), *Xanthoparmelia stenophylla* methanolic extract (XSM), and *Xanthoparmelia stenophylla* hexanic extract (XSH) are presented in Table 1.

The data in Table 1 show that the highest yields were obtained by extracting *X. stenophylla* lichen with acetone. This is not unusual, as acetone is most commonly used as a type of universal solvent in the extraction of various types of lichens [20]. The lowest yield was obtained when using n-hexane as a solvent, which indicates the lower presence of nonpolar components in the lichen itself.

### 2.2. Chromatographic Analysis of Extracts

**Figure 1:**
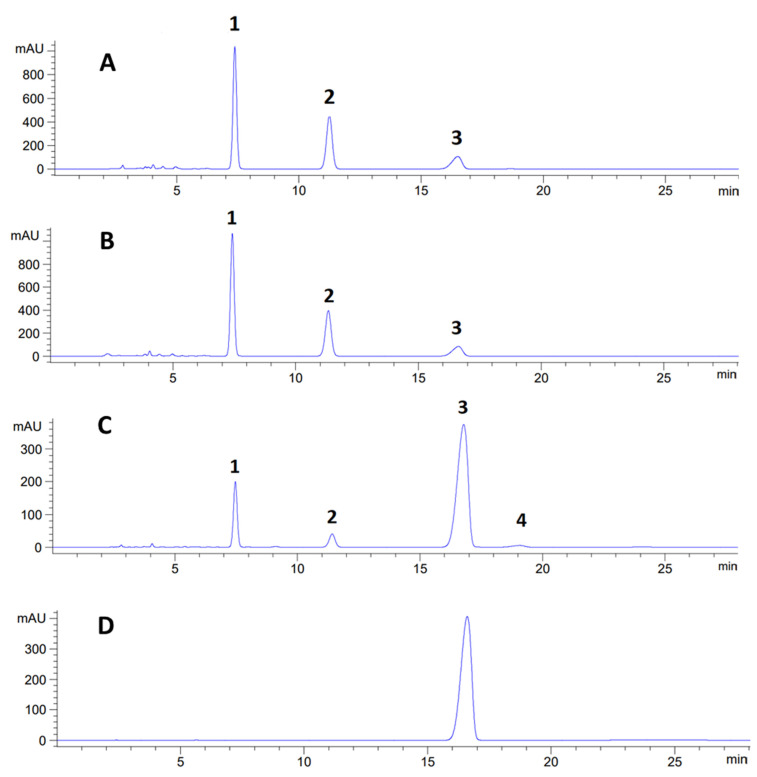
HPLC chromatograms of acetonic, methanolic, and hexanic extracts of lichen *Xanthoparmelia stenophylla* and the isolated compound usnic acid obtained at 254 nm. 1—Lecanoric acid; 2—obtusic acid; 3—usnic acid; 4—atranorin. (**A**) Chromatogram of *Xanthoparmelia stenophylla* acetonic extract obtained at 254 nm; (**B**) chromatogram of *Xanthoparmelia stenophylla* methanolic extract obtained at 254 nm; (**C**) chromatogram of *Xanthoparmelia stenophylla* hexanic extract obtained at 254 nm; (**D**) chromatogram of usnic acid obtained at 254 nm.

**Figure 2:**
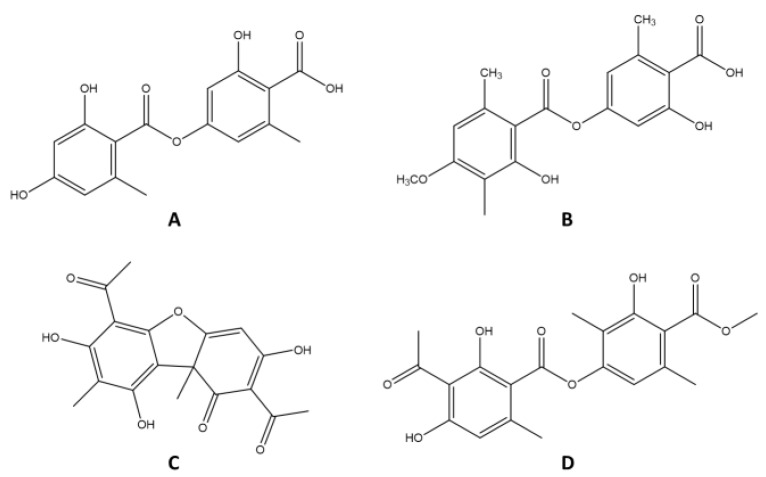
Chemical structures of compounds identified in the *Xanthoparmelia stenophylla* extracts: (**A**) lecanoric acid; (**B**) obtusic acid; (**C**) usnic acid; (**D**) atranorin.

Chromatograms for the tested extracts (i.e., XSA, XSM, and XSH), as well as for the isolated secondary metabolite (i.e., usnic acid), are presented in Figure 1. Based on the chromatograms, it can be seen that XSA and XSM had three dominant components (i.e., lecanoric acid, obtusic acid, and usnic acid), while the presence of atranorin as a fourth component was observed in XSH. Lecanoric acid, a compound of depsid structure, was identified in all three tested extracts at retention time t_R_ = 7.42 ± 0.02 but with different contents: XSA—4.0%, XSM—3.0%, and XSH—1.7%. Obtusic acid and atranorin had depside structures but with a different set of substituents. In XSH, the most common compound was usnic acid (t_R_ = 16.78 ± 0.02), with a characteristic dibenzofuran structures and a content of 58.7%. The presence of usnic acid has previously been documented in *X. stenophylla* lichen [17], while other components were identified for the first time. Detailed data on the retention time, absorption maxima, and content of the identified secondary metabolites are presented in Table 2, while the structures of the identified compounds are shown in Figure 2.

The presence of identified secondary metabolites was confirmed by comparing retention times and absorption maxima with standards obtained from the following sources: lecanoric acid (t_R_ = 7.42 ± 0.02) from lichen *Melanelia subaurifera*, obtusic acid (t_R_ = 11.41 ± 0.01) from lichen *Physcia semipinnata*, usnic acid (t_R_ = 16.78 ± 0.02) from lichen *Usnea barbata* and as a commercially available standard (Sigma Aldrich), and atranorin from lichen *Evernia prunastri*. All of the mentioned standards were obtained within the work of our laboratory [21,22,23], and their structures were confirmed based on spectral data. In addition, the limit of detection (LOD) and limit of quantification (LOQ) for the used chromatographic method and for every identified compound were determined (Table 3).

### 2.3. Total Phenolic Content and Total Flavonoid Content

The results of the total phenolic content (TPC) and total flavonoid content (TFC) are presented in Table 4. It can be observed that XSA had the highest values of TPC and TFC, both when quercetin (a flavonoid from the flavonol group) was used as the standard and when rutin (a flavonoid heteroside) was used as the standard. XSM had slightly lower values of TPC and TFC compared to XSA, while XSH had the least TPC and TFC. This was expected since phenolic compounds and flavonoids dissolve better in polar solvents such as acetone and methanol.

### 2.4. Antioxidant Activity

The results of testing the antioxidant activity of the analyzed extracts and usnic acid in the context of scavenging DPPH, hydroxyl, and superoxide anion radicals are presented in Table 5. The best antioxidant effect according to all three parameters showed XSA, then XSM, and finally XSH. Usnic acid performed better than any of the extracts but was still weaker compared to the positive controls (i.e., gallic acid and ascorbic acid).

### 2.5. Antimicrobial Activity

#### 2.5.1. Determination of Antimicrobial Activity

The results of in vitro antibacterial and antifungal activity of XSA, XSM, XSH, and usnic acid, as well as the positive controls (i.e., antibiotics: ampicillin and tetracycline); antifungals: fluconazole and amphotericin B), are shown in Table 6 and Table 7.

The antimicrobial activity of the examined extracts and usnic acid was determined against 19 species of microorganisms. In this experiment, the values of minimum inhibitory concentration (MIC) and minimum bactericidal concentration (MBC) were in a range of <0.08 to >10 mg/mL for XSA, XSM, XSH, and usnic acid and in range of <0.06 to >128 µg/mL for antibiotics. The intensity of the antibacterial activity depended on the type of extract and the species of microorganism. The extracts were active in the following ascending order: XSM < XSA < usnic acid < XSH.

Based on the results, it can be concluded that XSA had better antibacterial activity compared to XSM. Gram-positive bacteria showed higher susceptibility to the tested extracts. XSA showed no antibacterial activity against Gram-negative bacteria, i.e., *E. coli* ATCC 25922, *E. coli*, *S. enterica*, *S. typhimurium*, *K. pneumoniae* ATCC 70063, *K. pneumoniae*, and *S. aureus* (MIC and MBC > 10 mg/mL). MIC and MBC values for the rest of the tested bacteria ranged between 2.5 and 10 mg/mL. XSM showed no antibacterial activity against most of the tested bacteria. The exception was the MIC value of *Bacillus cereus* (5 mg/mL).

The antibacterial activity of XSH extract was mostly reflected in Gram-positive bacteria. *B. subtilis* ATCC 6633, *B. subtilis,* and *B. cereus* were highly sensitive to n-hexane extracts (MIC and MBC < 0.08 mg/mL), as well as *S. aureus* ATCC 6538 (MIC and MBC at 0.625 mg/mL) and *S. aureus* (MIC and MBC at 0.3125 mg/mL). Among the Gram-negative bacteria, XSH showed the best effect against *P. mirabilis* ATCC 12453, *P. mirabilis*, and *P. aeruginosa* ATCC 27853 (MIC at 5 mg/mL).

Usnic acid showed no effect against Gram-negative bacteria (MIC and MBC > 10 mg/mL), except for *P. aeruginosa* ATCC 27853 and *P. aeruginosa* (MIC at 10 mg/mL). However, the antibacterial activity against Gram-positive bacteria from the genus *Bacillus* was strong (MIC and MBC at <0.08 mg/mL), while the antibacterial activity against bacteria from the genus *Staphylococcus* ranged between 0.156 and 0.3125 mg/mL.

Based on the results and compared with the positive controls (i.e., tetracycline and ampicillin), the tested extracts and usnic acid showed limited (i.e., XSM and usnic acid) and moderate (i.e., XSA and XSH) effects. However, usnic acid and XSH showed a strong effect on the tested Gram-positive bacteria. It was observed that 10% DMSO did not inhibit the growth of microorganisms.

The antifungal activity of XSA and XSM was selective against the examined fungi (MIC and MFC at 10 mg/mL). The exception was the activity of XSA (MIC at 1.125 mg/mL) and the activity of XSM (MIC at 2.5 mg/mL) against *C. albicans* ATCC 10231. XSH showed the best antifungal activity against *C. albicans* ATCC 10231 (MIC at 0.625 mg/mL) and *A. niger* ATCC 16888 (MIC at 0.156 mg/mL). Usnic acid showed activity against the tested fungi at a concentration of 10 mg/mL, except against *S. cerevisiae* (MIC at 5 mg/mL). Generally speaking, XSH showed the best antifungal activity (Table 7).

#### 2.5.2. Antibiofilm Activity

In order to discover natural compounds capable of inhibiting and preventing bacterial biofilm formation, XSA, XSM, XSH, and usnic acid were examined. The antibiofilm effect of the extracts and compound on the *P. aeruginosa* and *S. aureus* ATCC 6538 biofilm formation was examined. The study demonstrated that the strains of bacteria formed a moderate biofilm in vitro (Table 8).

In the presence of the examined extracts, the possibility of biofilm formation was altered. Neither the tested extracts nor the usnic acid showed antibiofilm activity on *P. aeruginosa* (BIC_50_ and BIC_90_ > 10,000 µg/mL). The tested extracts and usnic acid showed an inhibitory effect against biofilm formation of *S. aureus* ATCC 6538 in the following ascending order: XSH < usnic acid < XSM < XSA.

**Figure 3:**
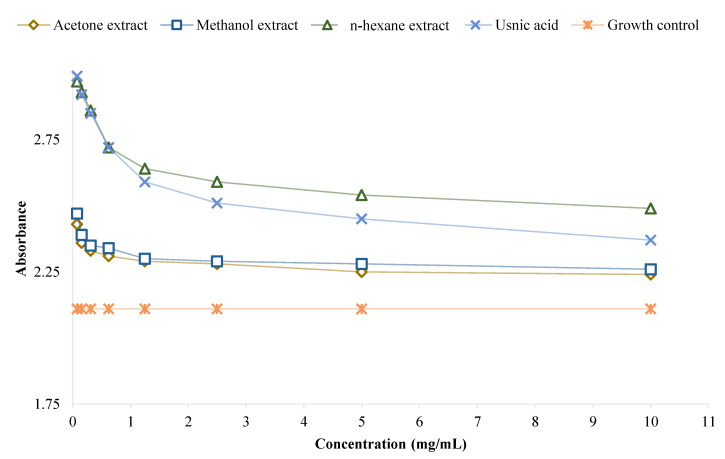
The impact of the tested extracts and usnic acid on the metabolic activity of *P. aeruginosa* biofilm.

**Figure 4:**
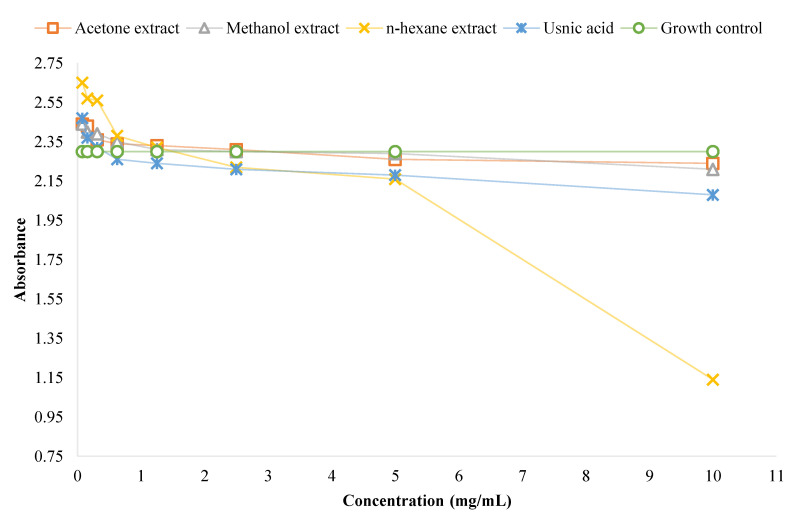
The impact of the tested extracts and usnic acid on the metabolic activity of *S. aureus* ATCC 6538 biofilm.

The biofilm metabolic activity of *P. aeruginosa* (Figure 3) and *S. aureus* ATCC 6538 (Figure 4) in the presence of the examined extracts and usnic acid were determined by adding resazurin.

The biofilm mass and metabolic activity results were correlated, i.e., an increase in biofilm mass productivity corresponded to increased biofilm metabolic activity. Briefly, the metabolic activity of *P. aeruginosa* biofilm was higher in the presence of the tested extracts and usnic acid compared to the growth control (Figure 3), while the metabolic activity of *S. aureus* ATCC 6538 biofilm was lower in the presence of the tested extracts and usnic acid compared to the growth control, at some concentrations (Figure 4).

### 2.6. Cytotoxic Activity

A panel of three different human cancer cell lines (i.e., MDA-MB 231, HeLa, and HCT-116) and a nontransformed cell line, MRC-5, were used in this study. Using the MTT colorimetric assay, the cytotoxic profile of the XSM, XSH, and XSA extracts as well as usnic acid (UA) were assessed. The results are represented as concentration–response curves and expressed as IC_50_, SI, GI_50_, TGI, and LC_50_ parameters.

The XSH and XSA extracts and UA demonstrated a statistically significant cell viability reduction in a concentration-dependent manner on each designated cell line after all observed time points (*p* < 0.05), with the exception of the XSH extract after 24 h of treatment. A time-dependent effect was observed only after treatment with XSH (*p* < 0.05). The XSM extract showed neither a concentration- nor time-dependent cell viability reduction. Dose–response curves of the MTT assay after 24, 48, and 72 h of treatment of MRC-5, MDA-MB 231 HeLa, and HCT 116 with XSM, XSH, XSA, UA, and doxorubicin are presented in Figures S1–S5.

The IC_50_ parameter (Table 9), as a measure of growth inhibition with no reference to the initial cell count, was determined to evaluate the overall inhibitory potential of the examined extracts. A varied susceptibility was observed within treated cell lines, namely, the XSA extract expressed the strongest overall inhibition against HeLa cells indicated by a low IC_50_ after 48 and 72 h of treatment, followed by the XSH extract’s moderate effect after 72 h of exposure, with a statistically significant difference. UA had a negligible effect (IC_50_ > 100 μg/mL). HCT-116 cells were equally and highly susceptible to the XSA extract and UA inhibitory effect after 72 h of treatment (*p* > 0.05), with no XSH extract effect observed. Moreover, there was no statistical difference between the XSA effect against HeLa and HCT 116 after 72 h of treatment. Overall, the inhibitory effect of the examined extracts and UA on MDA-MB 231 was of minor importance (data not shown). MRC-5 cells were negligibly susceptible to the XSH extract (IC_50_ > 100 μg/mL) and moderately sensitive to the XSA extract and UA with IC_50_ values of 51.63 ± 0.19 and 53.14 ± 1.04. The XSM extract showed no considerable effect on either tested cell line (data not shown). Compared to doxorubicin, none of the tested extracts or UA showed comparable overall inhibitory potential, whereas higher selective toxicity was observed. Selectivity for malignant cells relative to nontransformed MRC-5 cells was as the following: XSA extract was more selectively toxic for HeLa cells, with an SI value of 2.36, compared to the XSH extract with an SI lower than two, whereas the XSA extract and UA shared approximately equal selectivity for HCT-116 (i.e., 2.4 vs. 2.23).

Additionally, we assessed three parameters, considering a count of cells at time zero, to determine if the analyzed extracts exhibited cytostatic (i.e., GI_50_ and TGI) or cytocidal (i.e., LC_50_) effects on the designated cell lines (Table 10). None of the extracts examined exhibited cytocidal activity in any of the cell lines tested (LC_50_ > 100 μg/mL). Contrarily, in HeLa cells, the XSA extract elicited a robust antiproliferative effect with a 50% net cell growth inhibition at concentrations less than the lowest concentration investigated and a TGI value of 14.08 ± 2.54 μg/mL after 72 h of treatment. Moreover, XSA also displayed a high inhibition in HCT 116 net cell growth (GI_50_ < 0.1 μg/mL). In terms of total growth inhibition, XSA exhibited a significantly superior effect on HeLa cells compared to HCT 116 (*p* > 0.05). Additionally, MRC-5 cells were most prone to net cell growth inhibition of XSA compared to the XSH extract and UA. The XSH extract showed moderate and unsubstantial net cell and total growth inhibition, respectively, in HeLa and HCT 116 cells. UA displayed a high antiproliferative effect on respective HCT 116 cells.

## 3. Discussion

Our research showed that the lichen *X. stenophylla*, in addition to the already known usnic acid, also contains three compounds of depsid structure, namely, lecanoric acid and obtusic acid in all three tested extracts but with different contents, while atranorin was identified only in XSH. The highest TPC and TFC were detected in XSA. Moreover, XSA showed the best antioxidant and cytotoxic activity. On the other hand, XSH showed a higher antimicrobial potential in comparison with XSA and XSM.

Over the last decade, increasing research confirms the importance and role of oxidative stress in the development of cardiovascular [24,25], neurodegenerative [26,27], and reproductive diseases [28,29] as well as diseases associated with aging [30]. In addition, the role of oxidative stress in the development of lung damage after infection with the SARS-CoV-2 (COVID-19) virus has been observed [31,32]. Therefore, a large amount of research is aimed at discovering new antioxidant compounds, especially those from natural sources. Lichens are an important resource for detecting, identifying, and testing the effects of antioxidant compounds due to the fact of their secondary metabolites and their specific chemical structures characterized by a large number of free hydroxyl, carboxyl, phenolic, and keto groups, which can potentially be important for antioxidant activity [12,33]. Our research showed that XSA had the largest TPC and TFC, which is consistent with the solubility of this type of compound in polar solvents (such as acetone). The content of phenols and flavonoids in extracts can depend on many things such as the type of natural material for extraction, the solvent used, and the type of extraction [22,34]. In addition to having the highest TPC and TFC values, XSA exhibited the best antioxidant activity in all three systems used in this study (i.e., DPPH radical scavenging activity, hydroxyl radical scavenging activity, and superoxide anion radical scavenging activity). Although it has been shown that there is a positive correlation between the total content of phenolic compounds and antioxidant activity [12,33,35], it should always be assumed that the overall antioxidant effect depends not only on the phenolic compounds present but also on other compounds present in the extract, such as hydrates and proteins, which can act synergistically or, on the other hand, interact with the active centers of phenolic compounds and, thus, reduce their antioxidant activity [12,33,34]. Although there are numerous studies dealing with antioxidant compounds in general, as well as those focusing on the antioxidant potential of lichens and their secondary metabolites, the molecular mechanism by which these substances exhibit antioxidant activity is still unclear [12,33]. The depsid chemical structure of lecanoric acid has the potential to exert an antioxidant effect [21,36]. In our study, XSA showed the best antioxidant effect in all tests used. It should be noted that the acetonic extract had the highest flavonoid content expressed over mg QE/g dry extract and mg RE/g dry extract and also the highest content of lecanoric acid compared with the other two investigated extracts. It is possible that the compounds of flavonoid structure make up the largest part of the unidentified components of the acetone extract. The flavonoid structures present in lichen extracts appear to have a higher potential for antioxidant activity, which may explain the lower antioxidant activity of XSM and XSH compared to XSA. Atranorin present in XSH showed low to moderate antioxidant activity in previous studies [12,37]. Taking into account the prevalence and diversity of lichens, some secondary metabolites of lichens with significant antioxidant activity may be detected in the future.

In vitro evaluation of some lichens against human pathogenic bacteria was investigated [38,39]. The antimicrobial activity of *X. stenophylla* extracts was observed by some authors [40,41,42].

Kinalioğlu et al. [40] investigated the antimicrobial activity of acetone and chloroform extracts of *X. stenophylla*. They indicated that chloroform extract was the most effective against *Bacillus megaterium*. None of the tested extracts were effective against *K. pneumoniae*. In our study, the tested extracts showed no effect on *K. pneumoniae*, too. Simonyan et al. [42] indicated that methanolic, ethanolic, and acetonic extracts of *X. stenophylla* collected from Gegharkunik Province, Armenia, showed in vitro antibacterial activity against Gram-positive bacteria. In our study, XSA and XSM also showed a good effect on Gram-positive bacteria. Houshyar et al. [43] investigated the antimicrobial activity of acetone, methanolic, and chloroform extract of *X. stenophylla*. The results showed that the chloroform extract of *X. stenophylla* was much better than the others in the extraction of lichen antibacterial substances. The methanolic extract showed lower activity. In our study, XSA also showed a better antimicrobial effect than XSM. According to Bilgin Sökmen et al. [41], the acetonitrile extracts of the tested lichens showed maximum and minimum activity against *C. albicans* (32 mm) and *S. cerevisiae* (10 mm), respectively. The antifungal action of the lichen extracts increased in the following order: *F. caperata* > *X. conspersa* > *X. stenophylla*. In our study, XSH was the most effective against fungi. Therefore, lichen extract would be a promising biological product that would be a potential substitute instead of a chemical.

To the best of the authors’ knowledge, hexanic extract of *X. stenophylla* has not been examined so far. Therefore, this is the first time that the antimicrobial effects of hexanic extract of lichen *X. stenophylla* are presented. Based on HPLC analysis, it can be observed that the most dominant components in XSH are usnic acid and then lecanoric acid and atranorin, with a relatively small content of obtusic acid. The free carboxylic and phenolic groups in lecanoric acid can be potential carriers of antimicrobial activity [44], and usnic acid has already been shown to be a good antimicrobial agent in this paper and the available literature [19]. In one of our previous studies, it was shown that lecanoric acid has an approximately 10–20 times stronger effect compared to the extracts from which lecanoric acid was isolated [21]. Atranorin present in XSH is a moderate antimicrobial agent particularly effective against *S. aureus*, *B. subtilis*, *B. cereus*, *K. pneumonia*, and *E. coli* as well as against the fungi *C. albicans* and *A. niger* [37]. It is possible that the synergistic effect of lecanoric acid, usnic acid, and atranorin leads to the fact that XSH has a more pronounced antimicrobial activity compared to XSA and XSM but also in relation to usnic acid itself, especially in the strains *P. mirabilis* ATCC 12453, *P. mirabilis*, *K. pneumoniae*, and *P. aeruginosa* ATCC 27853. It was observed that the antimicrobial activity was connected to the concentration of secondary metabolites in the extracts.

Usnic acid, a compound produced by various lichen species, has an inhibitory effect on the growth of various bacteria. In a report by Maciazg-Dorszynska et al. [45], usnic acid caused rapid and strong inhibition of RNA and DNA synthesis in Gram-positive bacteria, represented by *B. subtilis* and *S. aureus*, while it did not inhibit the production of macromolecules (i.e., DNA, RNA, and proteins) in *E. coli*, which is resistant to even high doses of this compound. Inhibition of protein synthesis in *B. subtilis* and *S. aureus* was delayed, which suggests indirect action (possibly through impairment of transcription) of usnic acid on translation. Interestingly, DNA synthesis was halted rapidly in *B. subtilis* and *S. aureus*, suggesting interference of usnic acid with elongation of DNA replication. They propose that inhibition of RNA synthesis may be a general mechanism of antibacterial action of usnic acid, with additional direct mechanisms such as impairment of DNA replication in *B. subtilis* and *S. aureus*. According to our results, usnic acid has better activity on Gram-positive bacteria (MIC and MBC < 0.08 mg/mL), which is in accordance with the results of previous research [45].

To the best of the authors’ knowledge, the antibiofilm activity of the tested *X. stenophylla* extracts has not been investigated so far. Microorganisms that have the ability to adhere to the surface can form biofilms, and they are highly resistant to local or systemic infections. Currently, the evidence suggests that usnic acid, a secondary lichen metabolite, possesses antimicrobial activity against several planktonic Gram-positive bacteria including *S. aureus, Enterococcus faecalis*, and *E. faecium.* Francolini et al. [46] indicated that usnic acid may be utilized in the control of medical biofilms that are formed by *S. aureus* and *P. aeruginosa*. Usnic acid did not inhibit the initial attachment of *S. aureus* cells but killed the attached cells, which resulted in the inhibition of biofilm formation. Interestingly, although *P. aeruginosa* formed biofilm on the surface in the presence of usnic acid, the morphology of biofilm was altered, possibly indicating that usnic acid interfered with signaling pathways [46]. In our study, usnic acid showed an inhibitory effect on the biofilm formation of *S. aureus* (BIC_50_ at 1750 mg/mL), while no inhibitory effect on *P. aeruginosa* biofilm formation was detected.

It can be observed that although XSH showed the best antimicrobial activity, XSA showed the best antibiofilm activity. Even XSM showed better antibiofilm activity compared to XSH and usnic acid, which showed very weak antibiofilm activity in our experiments. This could mean that the main carriers of the antibiofilm activity of the tested extracts were not depsid metabolites of lichens (i.e., lecanoric acid, obtusic acid, and atranorin) and usnic acid but other present compounds. In addition, it should be noted that the tested extracts had an antibiofilm effect only against Gram-positive strain *S. aureus*, while not at all effective against Gram-negative strain *P. aeruginosa*. XSA contained the highest amounts of phenolic and flavonoid compounds compared to XSM and XSH.

The literature data do not provide an accurate explanation of the mechanism of antibiofilm activity of lichens and their extracts, but secondary lichen metabolites are generally thought to be responsible for antibiofilm activity as well as antimicrobial activity [47,48]. In a study by Pompilio et al. [48], the antibiofilm activity of usnic acid and atranorin, as pure substances, was examined, and it was shown that atranorin exhibited better action compared to usnic acid. A recent study showed that novel-type formulations such as usnic acid-impregnated graphene flakes could have a more prolonged antibiofilm activity [49]. In our study, XSH, the only extract that contained atranorin, showed the weakest antibiofilm effect compared to the other two tested extracts. To our knowledge, there are no studies that have individually examined the antibiofilm activity of lecanoric acid and obtusic acid. On the other hand, a study by Mitrovic et al. [47] showed that lichen extracts had a stronger antibiofilm effect against Gram-positive *S. aureus* than against Gram-negative *P. mirabilis*, which is comparable with our results. The results of Millot et al.’s [50] study showed that acetone extracts of lichens *R. fastigiata* and *E. prunastri* showed antibiofilm activity against fungus *C. albicans*, and squamatic acid, evernic acid, and stictic acid were identified as compounds with potentially the strongest antibiofilm activity. We did not examine antibiofilm activity against fungi, and this should certainly be the goal of future research.

The most frequently studied mechanism of bacterial adaptation to the environment, and thus responsible for biofilm formation, is the quorum-sensing mechanism (QS), which regulates communication between cells and is differently genetically regulated in Gram-positive and Gram-negative bacteria strains. In addition, the extracellular polymeric substance (EPS) is important, which is responsible for maintaining the structure of the biofilm and for protecting bacteria from the antimicrobial action of antibiotics [51,52]. Some previous studies have shown that some phenolic, and especially flavonoid compounds, exhibit antibiofilm activity against *S. aureus* using the same methodology as in our study [51,52], while Matilla-Cuenca et al. [53] examined antibiofilm activity of flavonoids towards *S. aureus* in more detail with the aim of discovering the mechanism of action. The anti-amyloid action of polyphenols (in which flavonoids can be classified) was recognized as the main mechanism of action. Flavonoids have been shown to inhibit biofilm associated protein (Bap)-mediated biofilm formation and, thus, prevent colonization.

A possible explanation for the difference in the action of different flavonoid compounds on the formation of biofilm of Gram-positive and Gram-negative bacteria is the diversity of flavonoid structures; therefore, flavones, flavonols, and anthocyanins have stronger activity against Gram-positive bacteria, while flavanones and flavanols have a stronger effect on Gram-negative bacteria, but it is certainly necessary to conduct additional research to identify all potential mechanisms of action and structure–activity relationships.

According to Chaves Simões et al. [54], the resazurin assay is the most sensitive method, able to detect lower numbers of active cells. Resazurin is a blue nonfluorescent dye (water-soluble, stable, nontoxic, and permeable through cell membranes) that is reduced to the pink-colored, highly fluorescent resorufin [55]. The resazurin assay was previously applied to determine the activity of biofilm cells as reported by Sarker et al. [56], while Pettit et al. [57] described it as a reliable and reproducible method for evaluating biofilm susceptibility to antimicrobials. The main advantage of the resazurin assay is that being a biological dye, it is nontoxic to cells. According to the results presented in this study, the results of the resazurin assay were correlated with the biofilm mass assay.

Various pharmacological remedies have been discovered using natural materials. Sixty percent of today’s medications are derived from natural compounds [58]. Many key commercial anticancer medications originate from natural sources. Improved cytotoxic agents are still being sought in the hunt for contemporary anticancer therapies [58], especially from unexplored natural sources such as lichens.

In this study, we examined the cytotoxic properties of XSA, XSM, XSH, and its secondary metabolite, usnic acid, on human breast cancer cells (i.e., MDA-MB 231), human cervix carcinoma cells (i.e., HeLa), and human colon cancer cells (i.e., HCT 116). To the best of our knowledge, this is the first report on the cytotoxic effect of *X. stenophylla* extracts against a tested panel of cell lines. We showed that XSA exhibited a concentration-dependent and the highest antiproliferative effect, with cervix and colon cancer cells being the most sensitive, whereas the cytocidal effect was not observed. XSM has been proven for its strong cytotoxicity against hepatocellular carcinoma cells (HepG2) and poorly differentiated colon carcinoma cells (RKO) [59], contrary to our results which reported no antitumor activity of XSM to any designated tumor cell lines in the tested concentration range. The most favorable antitumor effect of XSA against cervix and colon cancer cells, compared to other examined extracts, most probably derived from the optimal ratio of secondary metabolites and its synergism. HPLC analysis identified usnic acid and lecanoric acid as the main active compounds in this lichen, and the content of lecanoric acid was the highest in XSA. In recent research, lecanoric acid strongly reduced HeLa and HCT 116 cells’ viability and affected their proliferative capacity [60]. Usnic acid, as a common lichen secondary metabolite, present in all of our extracts, is one of few commercially available substances from lichens and is employed as such in more than half of publications concerning its anticancer properties [61]. In our research, usnic acid displayed a high impact on colon cancer cell proliferation capacity, with moderate and negligible effects on cervix and breast cancer cells, respectively. Other research referred to the extensive antiproliferative activity of synthetic derivatives of usnic acid against HeLa and MCF-7 cells [62]. On the other hand, XSH showed a less potent cytotoxic effect compared to XSA and usnic acid but more potent than XSM. The probable reason for these findings may be attributed to the presence of atranorin in XSH but not in XSM. In fact, atranorin as a pure substance showed a low to moderate cytotoxic effect, especially in the case of cell lines also used in our study [37]. Despite the fact that the observed cytotoxic activity of the XSA was lower than that of the positive control (i.e., doxorubicin), the current findings demonstrate that this lichen has higher selectivity against human cervix and colon tumors. Even though there has been much progress in the development of new anticancer drugs over the last few years, the large spectrum of side effects and general toxicity remains a stumbling stone to successful anticancer therapy. In common opinion, products derived from natural sources are considered less hazardous for normal cells, evidenced by a broad range of extracts and secondary metabolites that have been tested in clinical studies [63]. Therefore, finding new drugs from nature is still very important for modern cancer research, because many sources of natural products have not yet been investigated.

## 4. Materials and Methods

### 4.1. Lichen Sample

A sample of the lichen, *X. stenophylla*, from the family *Parmeliaceae* was collected in September 2021. The collection was carried out in the area of Stara Planina (GPS coordinates—43°21′51.9″ N 22°32′33.4″ E) in the eastern part of the Republic of Serbia. During the collection, a total of 160 g of lichen was collected, and care was taken not to endanger the survival of the lichen population at the place of collection. The lichen sample was deposited at the Department of Biology and Ecology of the Faculty of Science and Mathematics at the University of Nis (voucher number: 770NHM). Lichen determination was performed using standard techniques [64].

### 4.2. Preparation of Lichen Extracts

Extracts were prepared using the multiple maceration technique. The lichen material was dried at room temperature (22–25 °C) for 7 days with a daily change of the absorbent material below the lichen material. The dried lichen sample was ground to a fine powder using a mill (IKA A11, IKA^®^-Werke GmbH & Co., Staufen, Germany). After grinding, the powder was sieved through a sieve of 0.30 (according to Pharmacopoeia Jugoslavica IV [65]) which correlated to a particle size of 300 µm in order to obtain a uniform particle size of the whole sample and improve the extraction process. Solvents (i.e., acetone, methanol, and n-hexane, (Sigma Aldrich, St. Louis, MO, USA)) were prepared for extraction by redistilling and filtering through a membrane filter (MF-Millipore^®^ Membrane Filter, 0.22 μm pore size) for purification. Ground lichen (20 g) and 200 mL of each of the solvents were placed in three prepared glass bottles with a stopper. The bottles prepared in this way were left for 3 days in a dark place at room temperature with occasional shaking. The solvent was changed three times, and all fractions of the same solvent liquid extract were collected and filtered. The solvent was then evaporated using a rotary vacuum evaporator (IKA RV10, IKA^®^-Werke GmbH & Co., Staufen, Germany). Dry extracts were stored in a freezer (−18 °C) until analysis.

### 4.3. High-Performance Liquid Chromatography (HPLC) Analysis

Extracts were prepared for the HPLC analysis by dissolving in methanol (1 mL). A reverse-phase chromatographic column (C18; 25 cm × 4.6 mm; particle size 10 µm) and UV spectrophotometric detector were used for analysis on an Agilent 1200 System HPLC (Agilent Technologies, Santa Clara, CA, USA). The methanol–water–phosphoric acid solvent system (80:20:0.9, *v*/*v*/*v*) was used as the mobile phase, the flow rate was 1.0 mL/min, and the injected sample amount was 10 µL. This procedure was previously explained and used [34,66]. Experimental water was generated using a Milli-Q water purification system (Milford, MA, USA), methanol was HPLC grade (Merck, Darmstadt, Germany), and phosphoric acid was the analytical reagent grade (Sigma Aldrich). Chromatograms and UV spectral data were obtained at a wavelength of 254 nm.

#### Limit of Detection (LOD) and Limit of Quantification (LOQ)

LOD and LOQ were determined according to ICH guidelines and according to the method described in Saran et al. (2013) [67,68]. LOD was defined as the smallest amount of analyte that could be detected but not precisely determined, and LOQ was defined as the smallest amount of analyte that could be reliably quantified. The various increasing concentrations of the analyzed compounds (7 concentrations) were analyzed by the chromatographic technique described above, and the response areas of the peaks on the chromatogram were recorded. The LOD was calculated according to the formula: LOD = 10 * σ/k; the LOQ according to the formula: LOQ = 3.3 * σ/k, where σ stands for the standard deviation of the analyzed data group and k for the slope of the calibration curve. The LOD and LOQ were determined in the following range of concentrations: 1.56–100 µg/mL for obtusic acid and usnic acid, 7.81–500 µg/mL for lecanoric acid, and 0.78–50 µg/mL for atranorin. Calibration data are given in Table S1 of the Supplementary Materials.

### 4.4. Isolation of Usnic Acid

After HPLC analysis and confirmation of the presence of usnic acid in the tested extracts of the lichen *X. stenophylla*, isolation of usnic acid as one of the present secondary metabolites was performed. The principle of isolation has previously been successfully used in several studies [23]. Dry XSA (2000 mg) was dissolved in benzene and put at 4 °C to cool and form a precipitate. The precipitate formed was filtered and the residue on the filter paper was analyzed using the HPLC procedure described above. The HPLC analysis showed that the analyzed precipitate contained two components, namely, usnic acid and atranorin, in smaller quantities. In order to purify the sample, fractionation was performed on a chromatographic column (silica gel; below 0.063 mm; 230 mesh), using a mixture of ethyl acetate and cyclohexane (25:75, *v*/*v*) as the mobile phase. Usnic acid was the first eluted component, and after evaporation, recrystallization was performed in an ethanol–chloroform solvent system. In the end, 80 mg of pure substance was obtained, with a degree of purity of 98.8%. The identification of the substance was performed on the basis of the melting point, spectral data [64], and the comparison of the chromatogram with the chromatogram of the standard of usnic acid (Sigma Aldrich). The isolated substance was stored at −18 °C until analysis was performed.

### 4.5. Total Phenolic Content

The total amount of soluble phenolic compounds in the tested *X. stenophylla* lichen extracts was determined using Folin–Ciocalteu reagents according to the Slinkard and Singleton method [69] with slight modifications and using gallic acid as a standard. A series of standard gallic acid solutions (i.e., 1000, 500, 400, 200, 100, 50, and 25 µg/mL) was used to construct the calibration curve. Briefly, 50 μL of the test extract or standard was poured into a test tube, diluted with 150 μL of distilled water, and 1 mL of Folin–Ciocalteu reagent was added to the reaction mixture. The mixture was vortexed and allowed to incubate at room temperature for 5 min after which 800 μL of sodium carbonate (7.5%) was added. The tubes were then allowed to incubate for one hour in the dark with occasional shaking. The absorbance was measured at 760 nm, and the content of the phenolic compounds was expressed in mg of gallic acid equivalents per gram of dry extract (mg GAE/g dry extract). The stated parameter was obtained from the standard gallic acid curve and according to Equation (1):

Absorbance = 0.0026 * Total phenol + 0.0808 (R^2^ = 0.9978) (1)

### 4.6. Total Flavonoid Content

Determination of total flavonoid content was performed according to the method described in Sushant et al. [70] with modifications. This method is based on the reaction of flavonoids with AlCl_3_ to form a yellow-colored complex. Briefly, 200 µL of a 10% solution of AlCl_3_ in methanol, 200 μL of a 1M solution of potassium acetate, and 5.6 mL of distilled water were added to 1 mL of a solution of the tested extracts, isolated secondary metabolites, or standards of various concentrations (i.e., 1000, 500, 250, 125, 62.5, and 31.25 µg/mL). After incubation for 30 min at room temperature, absorbance at 415 nm was measured relative to the blank. Rutin and quercetin were used as standards, and the results are expressed in milligrams of quercetin equivalents and milligrams of rutin equivalents per gram of dry extract (mg QE/g dry extract or mg RE/g dry extract). After constructing the calibration curve, the following equations of the line were obtained: Equation (2) with quercetin as a standard and Equation (3) for rutin as a standard.

Absorbance = 0.0018 * Total flavonoid content + 0.0571 (R^2^ = 0.9986) (2)

Absorbance = 0.0011 * Total flavonoid content + 0.0268 (R^2^ = 0.9986) (3)

### 4.7. Antioxidant Activity

All chemicals for the antioxidant activity test were purchased from Sigma Aldrich. All measurements were conducted in triplicate, and a Shimadzu UV-1800 spectrophotometer was used for all measurements during antioxidant activity experiments. The IC_50_ value was used for comparing different radical scavenging effects between investigated extracts, isolated compounds, and positive controls.

#### 4.7.1. Radical Scavenging Activity

The ability to scavenge free radicals was tested using 1,1-diphenyl-2-picryl-hydrazyl (DPPH) according to the method described in Dorman et al. [71] with certain modifications. A solution of DPPH in methanol at a concentration of 0.05 mg/mL was first prepared. The solution was prepared ex tempore and stored in a tinted bottle in the refrigerator until the experiments were performed. After, a series of standard solutions of the tested extracts and usnic acid in methanol (i.e., 1000, 500, 250, 125, 62.5, and 31.25 µg/mL) were made. Into the flasks were poured 200 µL of the solution of the tested extracts or usnic acid of the stated concentrations and 2 mL of the DPPH solution. The reaction mixture was vortexed and allowed to incubate in the dark for one hour, after which the absorbance was measured at 517 nm relative to the blank. Ascorbic acid, gallic acid, and 6-hydroxy-2,5,7,8-tetramethylchroman-2-carboxylic acid (Trolox) (Acros Organics (Fair Lawn, NJ, USA)). were used as positive controls. The concentration of DPPH radicals was calculated according to Equation (4):

% inhibition = 100 * (Ac − As)/Ac(4)

Ac stands for absorbance of the control (which contains all reagents, except the tested extract or compound), and As is the absorbance of the sample.

Based on the obtained values, a nonlinear calibration curve was constructed and used to determine the concentration of the tested sample that inhibited 50% of the DPPH radicals (IC_50_) [72].

#### 4.7.2. Hydroxyl Radical Scavenging Activity

The ability to inhibit non-site-specific peroxidation mediated by hydroxyl radicals for XSA, XSM, and XSH was determined using the method previously described in the literature [73,74]. During the experiment, in a reaction mixture that contained 500 μL of 5.6 mM 2-deoxy-D-ribose in KH_2_PO_4_-NaOH buffer (50 mM, pH 7.4), 100 μL of 1.0 mM aqueous ascorbic acid, 100 μL of 1.0 mM H_2_O_2_, and 200 μL of premixed solution of 100 μM FeCl_3_ and 104 mM EDTA (1:1 *v*/*v*), 100 μL of extract dissolved in water was added. The tubes were vigorously shaken, vortexed, and incubated for 30 min at 50 °C. After incubation, 1 mL of 1% thiobarbituric acid (TBA) and 2.8% trichloroacetic acid (TCA) were added to each tube. The mixture was vortexed again and heated in a water bath for 30 min at 50 °C. The degree of oxidation of 2-deoxyribose was evaluated by determining the absorbance of the solution at 532 nm, and the percentage of inhibition was determined according to Equation (4).

Ac stands for absorbance of the control (which contains all reagents, except the tested extract or compound), and As is the absorbance of the sample.

Based on the obtained values, a nonlinear calibration curve was constructed and used to determine the concentration of the tested sample that inhibited 50% of the hydroxyl radicals (IC_50_) [72].

#### 4.7.3. Superoxide Anion Radical Scavenging Activity

The superoxide anion radical scavenging activity of the investigated extracts and usnic acid was determined by the method described by Nishimiki et al. [75] with modifications. To the reaction mixture consisting of 1 mL of nitroblue tetrazolium (NBT) solution (155 mM in 0.1 M phosphate buffer at pH 7.4) and 1 mL of nicotinamide adenine dinucleotide (NADH) solution (468 mM in 0.1 M phosphate buffer at pH 7.4) was added 0.1 mL of the test sample (i.e., 1000, 500, 250, 125, 62.5, and 31.25 µg/mL). To initiate the reaction, 0.1 mL of phenazine methosulfate (PMS) solution (60 mM in 0.1 M phosphate buffer at pH 7.4) was added to each tube. The mixture was incubated at room temperature for 5 min, and then the absorbance was measured at 560 nm relative to the blank. Ascorbic acid, gallic acid, and Trolox were used as positive controls, and a decrease in absorbance indicated increased superoxide anion radical scavenging activity. The percentage inhibition of superoxide anion generation was calculated based on Equation (5):

Superoxide anion scavenging activity (%) = 100 * (Ac − As)/Ac(5)

Ac stands for absorbance of the control (which contains all reagents, except the tested extract or compound), and As is the absorbance of the sample.

Based on the obtained values, a nonlinear calibration curve was constructed and used to determine the concentration of the tested sample that inhibited 50% of the superoxide anion radicals (IC_50_) [72].

### 4.8. In Vitro Antimicrobial Assay

#### 4.8.1. Test Substances and Test Microorganisms

The tested methanol, acetone, and n-hexane extracts, as well as the component (usnic acid) of *X. stenophylla*, were dissolved in 10% dimethyl sulfoxide (DMSO) and then diluted into the nutrient liquid medium. DMSO was obtained from Acros Organics (Fair Lawn, NJ, USA). Resazurin was obtained from Alfa Aesar GmbH & Co. (KG, Karlsruhe, Germany). An antibiotic, tetracycline (Pfizer Inc., New York, NY, USA), was dissolved in the nutrient liquid medium, a Mueller–Hinton broth (Torlak, Belgrade, Serbia), while antimycotic, fluconazole (Pfizer Inc., Brooklyn, NY, USA) was dissolved in Sabouard broth (Torlak, Belgrade, Serbia).

The antimicrobial activity of the extracts and components was tested against 19 microorganisms. The experiment involved 15 strains of bacteria (i.e., 6 standard strains: *Staphylococcus aureus* ATCC 6538, *Bacillus subtilis* ATCC 6633, *Proteus mirabilis* ATCC 12453, *Pseudomonas aeruginosa* ATCC 27853, *Klebsiella pneumoniae* ATCC 70063, and *Escherichia coli* ATCC 25922; 9 isolates: *Staphylococcus aureus*, *Escherichia coli*, *Salmonella enterica*, *Salmonella* Typhimurium, *Proteus mirabilis*, *Klebsiella pneumoniae*, *Pseudomonas aeruginosa*, *Bacillus subtilis*, and *Bacillus cereus*). In addition, four fungal strains (i.e., two standard strains: *Candida albicans* ATCC 10231 and *Aspergillus niger* ATCC 16888; two isolates: *Saccharomyces cerevisiae* and *Candida albicans*) were tested. All isolates were a generous gift from the Institute of Public Health, Kragujevac. The other microorganisms were provided from the collection held by the Microbiology Laboratory Faculty of Science, University of Kragujevac.

#### 4.8.2. Suspension Preparation

The bacterial suspensions were prepared by the direct colony method. The turbidity of the initial suspension was adjusted using a densitometer (DEN-1, BioSan, Riga, Latvia). When adjusted to the turbidity of the 0.5 McFarland’s standard (Andrews, 2005) the bacteria suspension contained approximately 10^8^ colony-forming units (CFU)/mL, and the suspension of yeast contained 10^6^ CFU/mL; 1:100 dilutions of the initial suspension were additionally prepared in sterile 0.85% saline. The suspensions of fungal spores were prepared by gentle stripping of spores from slopes with growing mycelia. The resulting suspensions were 1:1000 diluted in sterile 0.85% saline.

#### 4.8.3. Microdilution Method

Antimicrobial activity was tested by determining the minimum inhibitory concentrations (MICs) and minimum microbicidal concentrations (MBCs) using the microdilution plate method with resazurin [56]. The 96-well plates were prepared by dispensing 100 μL of Mueller–Hinton broth for bacteria and Sabouard broth for fungi into each well. A 10 μL aliquot from the stock solution of the tested extract or component was added to the first row of the plate. Then, two-fold serial dilutions were performed using a multichannel pipette. The obtained concentration range was from 10 to 0.08 mg/mL. The method is described in detail in [76].

Tetracycline, ampicillin, amphotericin B (Sigma Chemicals Co., Burlington, MA, USA), and fluconazole (Pfizer, New York, NY, USA) were used as a positive control; 10% DMSO (as solvent control test) was recorded not to inhibit the growth of microorganisms. Each test included growth control and sterility control. All the tests were performed in duplicate, and the MICs were constant.

Minimum microbicidal concentrations were determined by plating 10 μL of samples from wells where no indicator color change or no mycelia growth was recorded on a nutrient agar medium. At the end of the incubation period, the lowest concentration with no growth (no colony) was defined as the minimum microbicidal concentration.

### 4.9. Determination of Antibiofilm Activity

#### 4.9.1. Biofilm Formation of Tested Bacteria

The ability of *S. aureus* ATCC 6538 and *P. aeruginosa* to form biofilms was assayed as described by O’Toole et al. [77] with some modifications. Briefly, the tissue culture of 96-well microtiter plates (Sarstedt, Germany) were prepared by dispensing 100 µL of nutrient broth (i.e., Mueller–Hinton broth) into each well. Ten microliters of spore suspension (10^8^ colony-forming units (CFU)/mL) was added into each row. To promote biofilm formation, all plates were incubated at 37 °C/48 h. After incubation, the content of each well was removed and washed twice with 200 µL of sterile 0.85% saline to remove nonadherent and weakly adherent cells. Biofilms formed by adherent cells in 96-well microtiter plates were stained with crystal violet (0.1% *w*/*v*; Fluka AG, Buchs, Switzerland) and incubated at room temperature for 20 min. Excess stain was rinsed off by thorough washing with deionized water and then with 200 mL of 96% ethanol. Optical densities (ODs) of stained adherent bacteria were determined with an enzyme-linked immunosorbent assay (ELISA) plate reader (RT-2100C, Rayto, Shenzhen, China) at a 630 nm wavelength.

Only broth served as a control to check sterility and nonspecific binding of media. To compensate for background absorbance, OD readings from sterile medium, fixative, and dye were averaged and subtracted from all test values. All tests were performed in duplicate.

#### 4.9.2. Antibiofilm Activity of the Tested Extracts and Usnic Acid

The tissue culture of 96-well microtiter plates (Sarstedt, Nümbrecht, Germany) were prepared by dispensing 100 µL of nutrient broth (i.e., Mueller–Hinton broth) into each well. From the stock solution of the tested extracts or components (concentration: 20 mg/mL), 100 µL was added to the first row of the microtiter plate. Two-fold serial dilutions were then made using a multichannel pipette, following which 10 µL of fresh bacterial suspension was added to each well. The inoculated microtiter plates were incubated at 37 °C for 48 h. After incubation, the content of each well was gently removed by tapping the microtiter plates. The wells were washed with 200 mL of sterile 0.85% saline to remove free-floating bacteria. Biofilms formed by adherent cells in 96-well microtiter plates were stained with crystal violet (0.1% *w*/*v*; Fluka AG, Switzerland) and incubated at room temperature for 20 min. Excess stain was rinsed off by thorough washing with deionized water and then with 200 mL of 96% ethanol. The optical densities (ODs) of stained adherent bacteria were determined with an enzyme-linked immunosorbent assay (ELISA) plate reader at a 630 nm wavelength. The biofilm inhibitory concentration required to reduce biofilm coverage by 50% (BIC_50_) was defined as the lowest concentration of extract that showed a 50% inhibition on the biofilm formation [78].

Only broth or broth with extracts served as a control to check the sterility and nonspecific binding of media. To compensate for background absorbance, OD readings from sterile medium, extracts, fixative, and dye were averaged and subtracted from all test values. All tests were performed in duplicate. Tetracycline, vancomycin, and ceftriaxone dissolved in a nutrient liquid medium were used as the reference compounds.

#### 4.9.3. Biofilm Metabolic Activity Assessment by Resazurin

Resazurin (7-hydroxy-3H-phenoxazin-3-one-10-oxide) is a viability dye used to assess the metabolic activity of biofilms [54]. After incubation at 37 °C/48 h, a volume of 10 µL of resazurin indicator solution was added to each well. Plates were incubated in the dark for 3 h at 37 °C. The OD was measured at 590 nm using an enzyme-linked immunosorbent assay (ELISA) plate reader. In addition, the color change from purple to pink indicated the metabolic activity of the formed biofilm.

### 4.10. Cytotoxic Activity

#### 4.10.1. Cell Lines and Culture

Examination of the antitumor effects of the XSM, XSH, XSA extracts as well as UA was performed on human breast cancer (i.e., MDA-MB231), cervical adenocarcinoma (i.e., HeLa), and colon cancer (i.e., HCT-116) cell lines as well as on the nontransformed human lung fibroblast (i.e., MRC-5). Listed cell lines were obtained from American Type Culture Collection (ATCC).

The MDA-MB231, HeLa, HCT-116, and MRC-5 adherent cell lines were cultured as monolayers in the complete nutrient medium. All manipulations were performed in a biological safety cabinet level II A2 (Biobase Bioindustry, Shandong, China). A frozen cryotube with 1 mL of cell suspension, stored in liquid nitrogen, was thawed and the contents were transferred to a test tube with 9 mL of complete DMEM (Dulbecco’s modified Eagle’s medium) enriched with 10% heath-inactivated FBS (fetal bovine serum), L-glutamine (2 mM), nonessential amino acids (0,1 mM), penicillin (100 IU/mL), and streptomycin (100 μg/mL) and centrifuged for 15 min at 300× *g*. The supernatant was discharged, whereas the precipitated cells were resuspended in 5 mL of supplemented medium and transferred to a T-25 flask. The cells were grown in an incubator under standard culture conditions (37 °C, humidified air, and 5% CO_2_). By reaching subconfluency, the cells were passaged. First, the nutrient medium from the flask was removed and the cells were washed with PBS and treated with a combination of 0.5 mL of 0.25% trypsin and 0.05 mM EDTA in order to detach the cell layer. After 2 min in an incubator under standard conditions, trypsin was neutralized with 2.5 mL of complete DMEM, and 1 mL of this suspension was transferred to a new T-25 flask and filled up to a total of 5 mL of the supplemented DMEM.

#### 4.10.2. Extract Solutions

The XSM, XSH, XSA extracts, UA, and doxorubicin stock solutions were prepared as 100 mg/mL DMSO and stored at +4 °C. Treatment solutions of extract or doxorubicin in defined concentrations were made ex tempore by diluting stock solution in supplemented DMEM. The DMSO concentration in final solutions did not exceed 0.1%.

#### 4.10.3. MTT Assay

The effect of XSA, XSM, XSH, and usnic acid on the viability of MDA-MB231, HeLa, HCT116, and MRC-5 was examined by the MTT test described by Mosmann [79].

All cell lines were seeded in microtiter 96-well plates with a density of 3 × 10^3^ per well in all experiments and incubated overnight. After incubation, the supernatant was replaced with XSA, XSM, XSH, and usnic acid solutions in a range of seven different concentrations or supplemented medium (control). The final extract concentrations were 0.1, 0.3, 1, 3, 10, 30, and 100 μg/mL. Doxorubicin was used as a positive control in the same concentration range as the extracts. Following overnight incubation (time zero) and incubation periods of 24, 48, and 72 h, the supplemented medium or extract solutions were discarded, and the cells were treated with MTT solution (0.5 mg/mL in PBS) and incubated for at least two hours, after which the formazan crystals were dissolved by 150 µL DMSO per well. The plates were shaken for 5 min before being measured with a multiplate reader (MRC UT-2100c, Holon, Israel) at a 550 nm wavelength. All experiments were carried out at least three times in quadruplicate to ensure accuracy.

#### 4.10.4. Cytotoxicity Parameters

The MTT assay findings are expressed as a percentage of the values for control samples, which were arbitrarily set at 100%. The degree of cytotoxicity (C(%)) of the XSA, XSM, and XSH extracts as well as UA was calculated according to the formula C(%) = (A_0_−A) × 100/A_0_, with the absorbance of the control wells labeled as A_0_ and the absorbance after treatment as A. The parameter IC_50_ was used to determine the extract’s overall inhibitory activity, which was defined as the concentration of the agent that suppressed the biological activity of the target cells by 50%. The selectivity index (SI) was computed as the ratio of the IC_50_ values for the corresponding extracts for the nontransformed cell line and malignant cells. A selectivity index of less than 2 indicated that the extract expressed general toxicity, while extracts with an SI > 2 demonstrated selective toxicity, and those with an SI > 3 exhibited extremely selective toxicity [80].

The GI_50_, TGI, and LC_50_ values were computed for each extract in accordance with the guidelines of the National Cancer Institute (NCI). According to a simplified definition, GI_50_ was the concentration at which 100 × (T − T0)/(C − T0) = 50 and assessed the growth inhibitory potential of the tested extracts. In order to determine the cytostatic impact of a tested extract, the TGI value was calculated as the concentration of the tested extract at which 100 × (T − T0)/(C − T0) = 0. The LC_50_ value was the concentration of an extract at which 100 × (T − T0)/T0 = −50 was reached, and it was used to assess the cytotoxic impact of extracts on cells. In calculations for named parameters, T0 represents the absorbance of the test well at time zero (when the compound is added), T represents the absorbance of the test well after 24, 48, or 72 h of exposure to the test compound, and C represents the optical density of the control well. The value of parameters is indicated as higher or lesser than the highest or the lowest concentration tested if the desired impact was not achieved or exceeded.

The mean and standard deviation are used to represent the outcomes of the experiments. The free Microsoft Office Excel add-in ED50plus v1.0 software was obtained from http://www.sciencegateway.org/protocols/cellbio/drug/data/ (accessed on 30 March 2022) to compute the IC_50_, GI_50_, TGI, and LC_50_ values.

### 4.11. Statistical Analysis

All values are presented as the mean ± standard deviation for triplicate measurements. ANOVA analysis was used to compare the different groups. A value of *p* < 0.05 was considered significant. For IC_50_ determination, GraphPad Prism^®^ 8.0 (GraphPad Software, Inc., San Diego, CA, USA) was used. Microsoft Excel (Microsoft Excel^®^ version 2013, Microsoft Co., Ltd., Redmond, WA, USA) was used for generating graphs and calibration curves. All other statistical analyses were performed in IBM SPSS Statistics version 20 (IBM).

## 5. Conclusions

To the best of our knowledge, this was the first study to identify the presence of lecanoric acid, obtusic acid, and atranorin in *X. stenophylla* and also the first study to analyze the lichen *X. stenophylla* originating in the Balkan region. Bearing in mind the obtained results, the acetonic extract of the lichen *X. stenophylla* has the highest content of total phenols and flavonoids, exhibited the best antioxidant effect according to all analyzed parameters, exhibited the strongest antibiofilm activity against *S. aureus*, and had the strongest cytotoxic effect of the tested extracts. Therefore, our results can be a cornerstone for the development of new natural antioxidants and cancer drugs based on the *X. stenophylla* lichen.

On the other hand, hexanic extract of lichen *X. stenophylla* showed the best antimicrobial characteristics, especially against *B. subtilis*, *B. cereus*, and *S. aureus*, and the role of this extract in various inflammatory diseases should be investigated in future in vivo studies. Moreover, this research contributes to expanding the knowledge regarding the biodiversity, chemical composition, and biological activity of lichens originating from Serbia and the Balkans.

Despite the comprehensive results, some aspects remain unclear. The discrepancy between the results of antimicrobial activity and antibiofilm activity in the context of the weaker antibiofilm effect of XSH compared to XSA and XSM, although XSH showed the most potent antimicrobial effect, should be investigated in detail. It is still unclear whether the antibiofilm activity in the case of lichen extracts was derived from insufficiently investigated secondary metabolites or from other compounds of phenolic and flavonoid structure present in the extracts.

In order to determine additional pharmacological characteristics, as well as to clarify the mechanisms of action of various extracts of this lichen and its compounds, future studies on more complex cells and organisms are necessary.

## Figures and Tables

**Table 1:** Yield of the extraction of the lichen *Xanthoparmelia stenophylla* with different solvents.

Extract	Solvent	Mass of Dried Lichen (g)	Mass of Dry Extract (g)	Yield (%)
XSA	Acetone	20.00	1.01	5.05
XSM	Methanol	20.00	0.422	2.11
XSH	n-Hexane	20.00	0.182	0.91

XSA—Xanthoparmelia stenophylla acetonic extract; XSM—Xanthoparmelia stenophylla methanolic extract; XSH—Xanthoparmelia stenophylla hexanic extract.

**Table 2:** Retention time, absorbance maxima, and content of the identified lichen substances.

Compound	Retention Time (t_R_ ± SD) (min)	Absorbance Maxima (nm)	Content (mg/g Dry Extract)		
			Extract		
			XSA	XSM	XSH
Lecanoric acid	7.42 ± 0.02	213, 270, 304	39.96	29.68	17.45
Obtusic acid	11.41 ± 0.01	212, 278, 312	24.96	28.87	2.61
Usnic acid	16.78 ± 0.02	234, 282	44.47	48.52	587.33
Atranorin	19.07 ± 0.01	210, 252, 262, 320	ND	ND	15.25

XSA—*Xanthoparmelia stenophylla* acetonic extract; XSM—*Xanthoparmelia stenophylla* methanolic extract; XSH—*Xanthoparmelia stenophylla* hexanic extract; ND—not detected. The values of the retention times are expressed as the means ± SD of three measurements.

**Table 3:** Linearity, range, the limit of detection, and the limit of quantification of the used chromatographic method for compounds identified in lichen *Xanthoparmelia stenophylla*.

Compound	Equation	Linearity (R^2^)	Linear Range (µg/mL)	LOD (µg/mL)	LOQ (µg/mL)
Lecanoric acid	Y = 20.435 ∗ X − 6.494	0.9998	7.81–500	3.025	9.167
Obtusic acid	Y = 11.502 ∗ X − 2.052	0.9993	1.56–100	0.590	1.787
Usnic acid	Y = 2.468 ∗ X + 0.233	0.9999	1.56–100	1.177	3.566
Atranorin	Y = 3.175 ∗ X + 1.569	0.9998	0.78–50	1.010	3.061

R^2^—Correlation coefficient of calibration curve; LOD—limit of detection; LOQ—limit of quantification.

**Table 4:** Total phenolic content and total flavonoid content of different *Xanthoparmelia stenophylla* extracts.

Extract	Total Phenolic Content (mg GAE/g Dry Extract)	Total Flavonoid Content (mg QE/g Dry Extract)	Total Flavonoid Content (mg RE/g Dry Extract)
XSA	167.03 ± 1.12 ^a^	178.84 ± 0.93 ^a^	320.18 ± 1.86 ^a^
XSM	94.70 ± 0.86 ^a,b^	116.06 ±0.31 ^a,b^	217.46 ± 1.34 ^a,b^
XSH	34.31 ± 0.52 ^a,b^	73.28 ± 0.29 ^a,b^	147.45 ± 1.02 ^a,b^

GAE—Gallic acid equivalent; QE—quercetin equivalent; RE—rutin equivalent; XSA—*Xanthoparmelia stenophylla* acetonic extract; XSM—*Xanthoparmelia stenophylla* methanolic extract; XSH—*Xanthoparmelia stenophylla* hexanic extract. All measurements were conducted in triplicate. The results are presented as the mean ± standard deviation (for three measurements). ^a,b^ Sharing of the same letter denotes groups significantly different at the level of *p* < 0.05.

**Table 5:** The antioxidant activity of different *Xanthoparmelia stenophylla* extracts and usnic acid.

Extract/Compound	DPPH Radical Scavenging Activity (IC_50_ (µg/mL))	Hydroxyl Radical Scavenging Activity (IC_50_ (µg/mL))	Superoxide Anion Radical Scavenging Activity (IC_50_ (µg/mL))
XSA	81.22 ± 0.54 ^a^	134.48 ± 0.76 ^a^	107.39 ± 0.58 ^a^
XSM	89.47 ± 0.66 ^b^	145.75 ±0.61 ^b^	116.53 ± 0.74 ^b^
XSH	95.55 ± 0.45 ^a,b,c^	154.16 ± 0.69 ^a,c^	125.16 ± 0.55 ^a,c^
Usnic acid	64.92 ± 1.06 ^a,b,c,d^	120.05 ± 0.85 ^a,b,c,d^	90.32 ± 0.95 ^a,b,c,d^
Gallic acid	3.72 ± 0.65 ^a,b,c,d,e^	45.12 ± 0.51 ^a,b,c,d,e^	68.55 ± 0.45 ^a,b,c,d,e^
Ascorbic acid	6.31 ± 0.78 ^a,b,c,d^	155.91 ± 0.64 ^a,d,e^	105.62 ± 0.84 ^c,d,e,f^
Trolox	10.79 ± 0.83 ^a,b,c,d,e^	131.45 ± 0.56 ^c,e^	135.14 ± 0.88 ^a,b,d,e,f^

XSA—*Xanthoparmelia stenophylla* acetonic extract; XSM—*Xanthoparmelia stenophylla* methanolic extract; XSH—*Xanthoparmelia stenophylla* hexanic extract. IC_50_ values were obtained using nonlinear regression analysis. All measurements were conducted in triplicate. The results are presented as the mean ± standard deviation (for three measurements); ^a,b,c,d,e,f^ Sharing of the same letter denotes groups significantly different at the level of *p* < 0.05.

**Table 6:** Antibacterial activity of the acetonic, methanolic, and hexanic extracts of lichen *X. stenophylla* as well as usnic acid.

Extracts/Compound	Gram Sign	XSA		XSM		XSH		UA		TC		AMP	
Bacterial species		MIC	MBC	MIC	MBC	MIC	MBC	MIC	MBC	MIC	MBC	MIC	MBC
*E. coli* ATCC 25922	G−	<10	<10	<10	<10	10	10	<10	<10	4	6	0.37	0.5
*E. coli*	G−	<10	<10	<10	<10	10	<10	<10	<10	2	6	2.1	1.2
*S. enterica*	G−	<10	<10	<10	<10	10	10	<10	<10	2	2	1	1
*S.* Typhimurium	G−	<10	<10	10	10	10	10	<10	<10	2	2	2	2
*P. mirabilis* ATCC 12453	G−	5	5	10	<10	5	7.5	<10	<10	2	4	4	4
*P. mirabilis*	G−	10	10	10	10	5	5	<10	<10	>128	>128	>128	>128
*K. pneumoniae* ATCC 70063	G−	<10	<10	<10	<10	10	<10	<10	<10	4	64	>128	>128
*K. pneumoniae*	G−	<10	<10	<10	<10	7.5	10	<10	<10	4	32	>128	>128
*P. aeruginosa* ATCC 27853	G−	2.5	10	10	<10	5	10	10	<10	4	32	>128	>128
*P. aeruginosa*	G−	5	10	10	<10	10	10	10	<10	>128	>128	>128	>128
*B. subtilis* ATCC 6633	G+	10	<10	<10	<10	<0.08	<0.08	<0.08	<0.08	0.25	0.37	3	4
*B. subtilis*	G+	5	10	10	<10	<0.08	<0.08	<0.08	<0.08	<0.06	0.25	16	128
*B. cereus*	G+	2.5	10	5	<10	<0.08	<0.08	<0.08	<0.08	0.25	0.5	4	6
*S. aureus* ATCC 6538	G+	10	<10	<10	<10	0.625	0.625	0.3125	0.625	3	6	0.25	0.75
*S. aureus*	G+	<10	<10	<10	<10	0.3125	0.3125	0.156	0.156	<0.06	<0.06	<0.06	<0.06

XSA—*Xanthoparmelia stenophylla* acetonic extract; XSM—*Xanthoparmelia stenophylla* methanolic extract; XSH—*Xanthoparmelia stenophylla* hexanic extract; UA—usnic acid; TC—tetracycline; AMP—ampicillin; MIC—minimum inhibitory concentration; MBC—minimum bactericidal concentration; G−—Gram-negative strain; G+—Gram-positive strain. Data are given in mg/mL for lichen extracts and in µg/mL for antibiotics.

**Table 7:** Antifungal activity of acetonic, methanolic, and hexanic extracts of the lichen *X. stenophylla* as well as usnic acid.

Extracts/Compound	XSA		XSM		XSH		UA		AB		FC	
Fungal Species	MIC	MFC	MIC	MFC	MIC	MFC	MIC	MFC	MIC	MFC	MIC	MFC
*S. cerevisiae*	10	10	10	10	1.25	2.5	5	10	/	/	7.81	7.81
*C. albicans* ATCC 10231	1.125	5	2.5	10	0.625	5	10	<10	0.49	1.95	31.25	31.25
*C. albicans*	10	10	10	10	1.25	5	10	<10	0.98	1.95	62.5	62.5
*A. niger* ATCC 16888	10	10	10	10	0.156	2.5	10	<10	0.98	0.98	250	500

XSA—*Xanthoparmelia stenophylla* acetonic extract; XSM—*Xanthoparmelia stenophylla* methanolic extract; XSH—*Xanthoparmelia stenophylla* hexanic extract; UA—usnic acid; AB—amphotericin B; FC—fluconazole; MIC—minimum inhibitory concentration; MFC—minimum fungicidal concentration. Data are given in mg/mL for lichen extracts and in µg/mL for antimycotics. /—not investigated.

**Table 8:** The influence of acetonic, methanolic, and hexanic extracts as well as usnic acid of *X. stenophylla* on biofilm formation.

Extract/Compound	Level of BIC	Bacterial Species	
		*P. aeruginosa*	*S. aureus* ATCC 6538
XSA	BIC_50_	<10,000	156
	BIC_90_	<10,000	1250
XSM	BIC_50_	<10,000	234
	BIC_90_	<10,000	2500
XSH	BIC_50_	<10,000	1250
	BIC_90_	<10,000	3750
UA	BIC_50_	<10,000	1750
	BIC_90_	<10,000	5000
TC	BIC_50_	>1000	249
	BIC_90_	>1000	>1000
VM	BIC_50_	733.8	62.6
	BIC_90_	>1000	>1000
CT	BIC_50_	116.2	475.4
	BIC_90_	>1000	>1000

XSA—*Xanthoparmelia stenophylla* acetonic extract; XSM—*Xanthoparmelia stenophylla* methanolic extract; XSH—*Xanthoparmelia stenophylla* hexanic extract; UA—usnic acid; TC—tetracycline; VM—vancomycin; CT—ceftriaxone; BIC—biofilm inhibitory concentration; BIC_50_—biofilm inhibitory concentration required to reduce biofilm coverage by 50%; BIC_90_—biofilm inhibitory concentration required to reduce biofilm coverage by 90%. Data are given in µg/mL.

**Table 9:** IC_50_ (μg/mL) values determined by MTT assay for XSA, XSH, UA, and doxorubicin after 24, 48, and 72 h of treatment of MRC-5, HeLa, and HCT 116 cells and SI values.

Tested Extract/Compound		XSA		XSH		UA		DOX	
Cell Type	Time	IC_50_	SI	IC_50_	SI	IC_50_	SI	IC_50_	SI
**MRC-5**									
	24 h	>100	NA	>100	NA	66.18 ± 1.72	NA	86.87 ± 17.17	NA
	48 h	52.96 ± 2.48	NA	>100	NA	78.54 ± 0.87	NA	0.96 ± 0.10	NA
	72 h	51.63 ± 0.19	NA	>100	NA	53.14 ± 1.04	NA	0.1 ± 0.05	NA
**HeLa**									
	24 h	>100	0.51	>100	0.94	>100	0.36	22.92 ± 3.05	3.8
	48 h	32.31 ± 3.14	1.63	>100	1.29	>100	0.41	1.04 ± 0.06	0.92
	72 h	21.17 ± 1.85	2.36	51.37 ± 0.69	1.95	>100	0.38	0.14 ± 0.01	0.71
**HCT 116**									
	24 h	>100	0.68	>100	1.52	>100	0.62	152.92 ± 9.69	0.57
	48 h	28.45 ± 0.17	1.86	>100	1.24	83.99 ± 4.22	0.93	126.04 ± 8.96	1.09
	72 h	21.48 ± 3.55	2.40	87.31 ± 1.44	1.03	23.79 ± 2.10	2.23	0.41 ± 0.16	0.24

XSA—*Xanthoparmelia stenophylla* acetonic extract; XSH—*Xanthoparmelia stenophylla* hexanic extract; UA—usnic acid; DOX—doxorubicin; SI—selectivity index; NA—not applicable. The results are presented as the mean ± standard deviation (for three measurements).

**Table 10:** XSA, XSH, UA, and doxorubicin concentrations (µg/mL) were needed for 50% growth inhibition (GI_50_) and total growth inhibition (TGI) of MRC-5, HeLa, and HCT 116 cells after 24, 48, and 72 h treatment.

Tested Extract/Compound		XSA		XSH		UA		DOX	
Cell Type	Time	GI_50_	TGI	GI_50_	TGI	GI_50_	TGI	GI_50_	TGI
**MRC-5**									
	24 h	37.73 ± 0.54	89.12 ± 0.44	>100	>100	42.97 ± 1.56	>100	<0.1	54.71 ± 4.65
	48 h	30.79 ± 1.22	75.81 ± 0.58	91.02 ± 11.06	>100	41.31 ± 0.56	93.25 ± 0.08	<0.1	6.34 ± 2.90
	72 h	11.38 ± 5.76	69.62 ± 7.70	60.04 ± 1.57	>100	36.73 ± 0.92	89.15 ± 0.25	<0.1	2.35 ± 0.35
**HeLa**									
	24 h	38.43 ± 2.21	84.05 ± 11.17	67.83 ± 9.93	>100	>100	>100	<0.1	<0.1
	48 h	11.48 ± 5.64	57.31 ± 3.34	51.41 ± 5.41	>100	99.16 ± 15.59	>100	<0.1	<0.1
	72 h	<0.1	14.08 ± 2.54	39.24 ± 0.83	91.38 ± 0.22	64.28 ± 8.34	86.91 ± 5.42	<0.1	<0.1
**HCT 116**									
	24 h	27.24 ± 0.45	88.98 ± 0.51	68.79 ± 18.15	>100	41.94 ± 3.12	79.12 ± 1.18	<0.1	<0.1
	48 h	17.35 ± 2.52	75.92 ± 0.15	61.4 ± 3.53	>100	27.21 ± 1.94	63.58 ± 0.90	<0.1	<0.1
	72 h	<0.1	55.65 ± 4.49	51.89 ± 0.15	99.48 ± 3.45	15.87 ± 2.89	64.91 ± 1.44	<0.1	<0.1

XSA—*Xanthoparmelia stenophylla* acetonic extract; XSH—*Xanthoparmelia stenophylla* hexanic extract; UA—usnic acid; DOX—doxorubicin; SI—selectivity index. The results are presented as the mean ± standard deviation (for three measurements).

## Data Availability

The data presented in this study are available in this article and the Supplementary Materials.

## Supplementary Materials

The following supporting information can be downloaded at: https://www.mdpi.com/article/10.3390/plants11131624/s1, Figure S1: Dose-response curves of MTT assay after 24, 48 and 72 h treatment of MRC-5, MDA-MB 231 HeLa and HCT 116 with XSM (*Xanthoparmelia stenophylla* methanolic extract). The values are presented as mean ± SD of quadriplicates from at least three independent experiments.; Figure S2: Dose-response curves of MTT assay after 24, 48 and 72 h treatment of MRC-5, MDA-MB 231 HeLa and HCT 116 with XSH (*Xanthoparmelia stenophylla* hexanic extract). The values are presented as mean ± SD of quadriplicates from at least three independent experiments.; Figure S3: Dose-response curves of MTT assay after 24, 48 and 72 h treatment of MRC-5, MDA-MB 231 HeLa and HCT 116 with XSA (*Xanthoparmelia stenophylla* acetonic extract). The values are presented as mean ± SD of quadriplicates from at least three independent experiments.; Figure S4: Dose-response curves of MTT assay after 24, 48 and 72 h treatment of MRC-5, MDA-MB 231 HeLa and HCT 116 with usnic acid. The values are presented as mean ± SD of quadriplicates from at least three independent experiments.; Figure S5: Dose-response curves of MTT assay after 24, 48 and 72 h treatment of MRC-5, MDA-MB 231 HeLa and HCT 116 with doxorubicin. The values are presented as mean ± SD of quadriplicates from at least three independent experiments.;Table S1: Values used for the calculation of limit of detection (LOD) and limit of quantification (LOQ).
