# Supplementary material for: Phytochemical Analysis, Antioxidant, Antimicrobial, and Cytotoxic Activity of Different Extracts of Xanthoparmelia stenophylla Lichen from Stara Planina, Serbia
Source: Plants (Basel). 2022 Jun 21;11(13):1624. doi: 10.3390/plants11131624 (PMC9269301; doi:10.3390/plants11131624)
Supplement: Supplementary file 1 [file plants-11-01624-s001.zip › plants-1685370-supplementary.pdf]

Supplementary Material

Phytochemical Analysis, Antioxidant, Antimicrobial, and Cytotoxic Activity of Different Extracts of *Xanthoparmelia stenophylla* Lichen from Stara Planina, Serbia

Aleksandar Kocovic [1], Jovana Jeremic [1,*], Jovana Bradic [1], Miroslav Sovrlic [1], Jovica Tomovic [1], Perica Vasiljevic [2], Marijana Andjic [1], Nevena Draginic [1,3], Mirjana Grujovic [4], Katarina Mladenovic [4], Dejan Baskic [5,6], Suzana Popovic [5], Sanja Matic [1], Vladimir Zivkovic [7], Nevena Jeremic [1,8], Vladimir Jakovljevic [3,7] and Nedeljko Manojlovic [1]

[1] Department of Pharmacy, Faculty of Medical Sciences, University of Kragujevac, 34000 Kragujevac, Serbia; salekkg91@gmail.com (A.K.); jovanabradickg@gmail.com (J.B.); sofke-ph@hotmail.com (M.S.); jovicatomovic2011@gmail.com (J.T.); andjicmarijana10@gmail.com (M.A.); nevenasdraginic@gmail.com (N.D.); sanjad.matic@gmail.com (S.M.); nbarudzic@hotmail.com (N.J.); mtnedeljko@gmail.com (N.M.)

[2] Department of Biology and Ecology, Faculty of Sciences and Mathematics, University of Niš, 18000 Niš, Serbia; pericavasiljevic@gmail.com

[3] Department of Human Pathology, 1st Moscow State Medical University IM Sechenov, 119991 Moscow, Russia; drvladakgbg@yahoo.com

[4] Department of Science, Institute for Information Technologies, University of Kragujevac, 34000 Kragujevac, Serbia; mirjana.grujovic@pmf.kg.ac.rs (M.G.); katarina.mladenovic@pmf.kg.ac.rs (K.M.)

[5] Centre for Molecular Medicine and Stem Cell Research, Faculty of Medical Sciences, University of Kragujevac, 34000 Kragujevac, Serbia; dejan.baskic@gmail.com (D.B.); popovic007@yahoo.com (S.P.)

[6] Institute of Public Health Kragujevac, 34000 Kragujevac, Serbia

[7] Department of Physiology, Faculty of Medical Sciences, University of Kragujevac, 34000 Kragujevac, Serbia; vladimirziv@gmail.com

[8] Faculty of Pharmacy, IM Sechenov First Moscow State Medical University (Sechenov University), 119991 Moscow, Russia

* Correspondence: jovana.jeremic@medf.kg.ac.rs; Tel.: +381-3430-6800

Table S1. Values used for the calculation of limit of detection (LOD) and limit of quantification (LOQ).

Compound	The slope of the calibration curve (k)	The standard deviation of the response (σ)	Limit of detection (LOD)= 3.3σ/k	Limit of quantification (LOQ) = 10σ/k
Lecanoric acid	20.435109	18.732702	3.025	9.167
Obtusic acid	11.501667	2.055429	0.590	1.787
Usnic acid	2.468396	0.880237	1.177	3.566
Atranorin	3.174977	0.971813	1.010	3.061

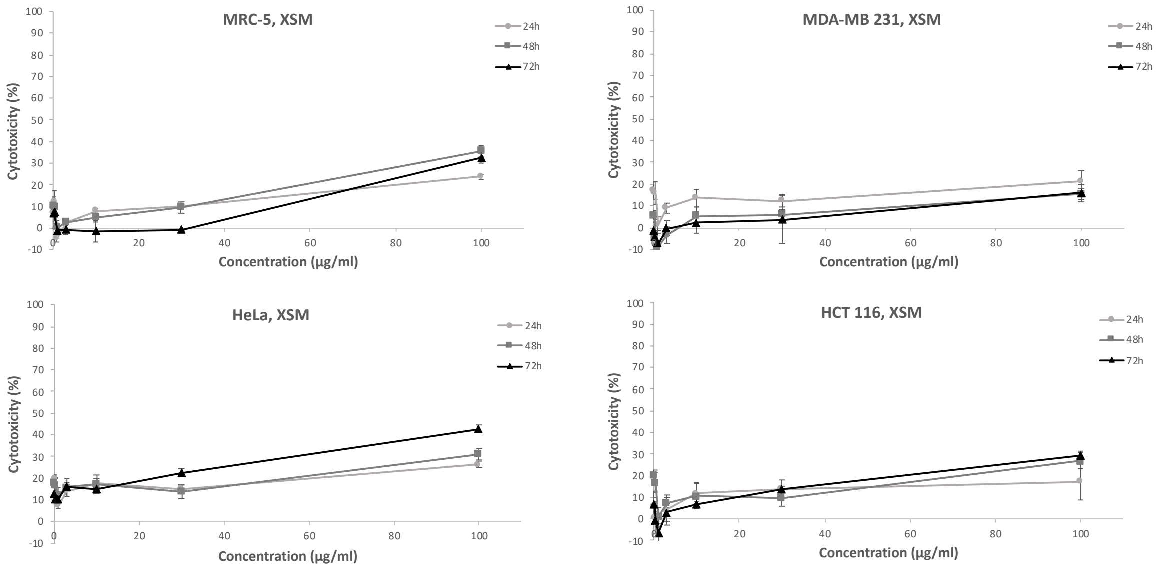

Figure S1. Dose-response curves of MTT assay after 24, 48 and 72h treatment of MRC-5, MDA-MB 231 HeLa and HCT 116 with XSM (*Xanthoparmelia stenophylla* methanolic extract). The values are presented as mean ± SD of quadriplicates from at least three independent experiments.

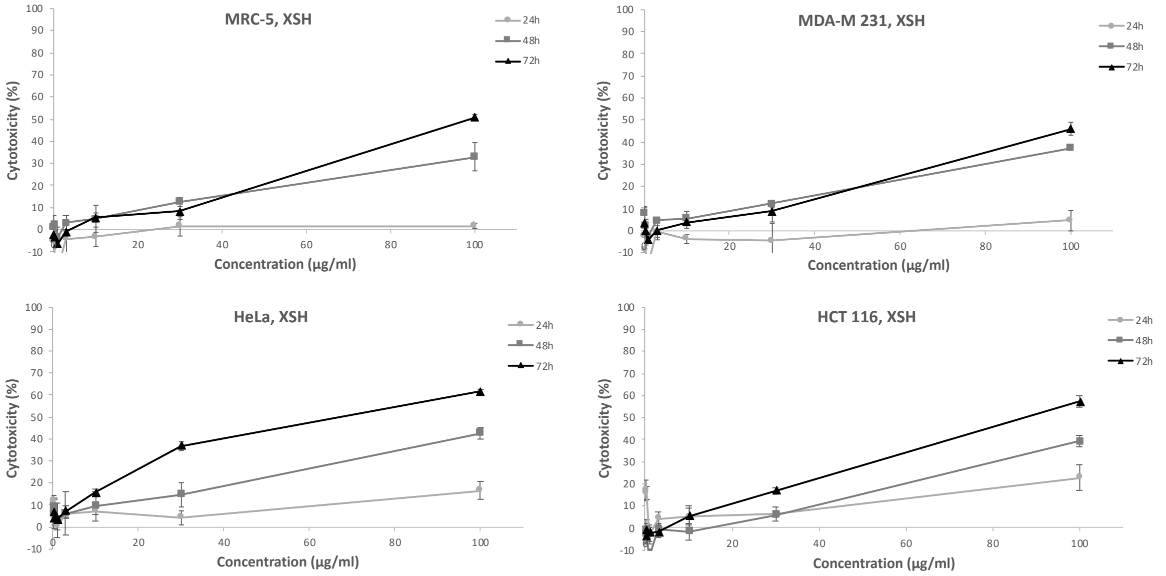

Figure S2. Dose-response curves of MTT assay after 24, 48 and 72h treatment of MRC-5, MDA-MB 231 HeLa and HCT 116 with XSH (*Xanthoparmelia stenophylla* hexanic extract). The values are presented as mean ± SD of quadriplicates from at least three independent experiments.

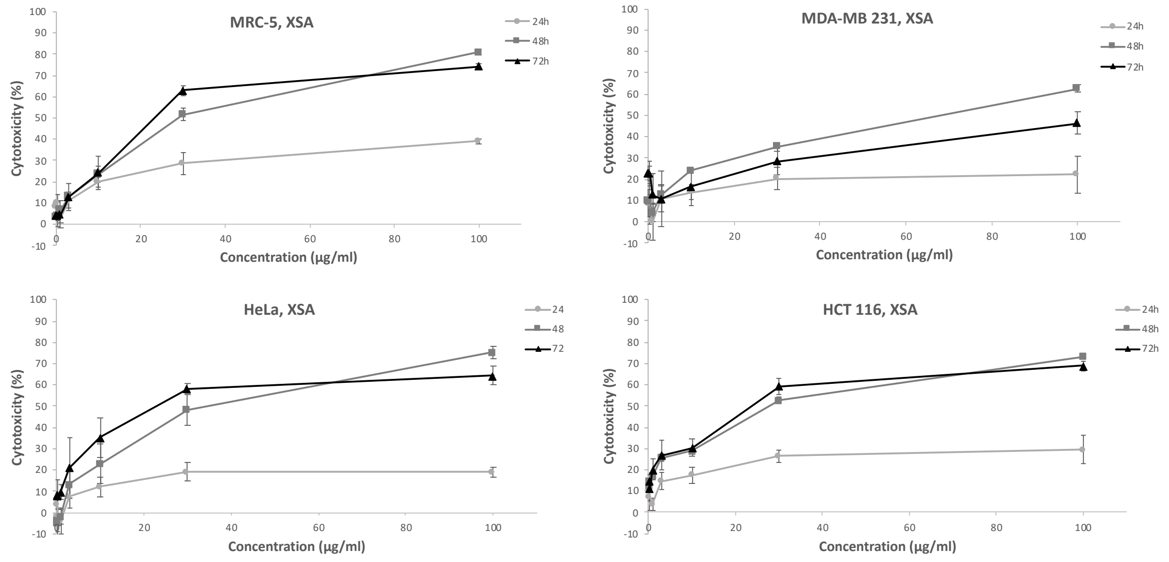

Figure S3. Dose-response curves of MTT assay after 24, 48 and 72h treatment of MRC-5, MDA-MB 231 HeLa and HCT 116 with XSA (*Xanthoparmelia stenophylla* acetonic extract). The values are presented as mean ± SD of quadriplicates from at least three independent experiments.

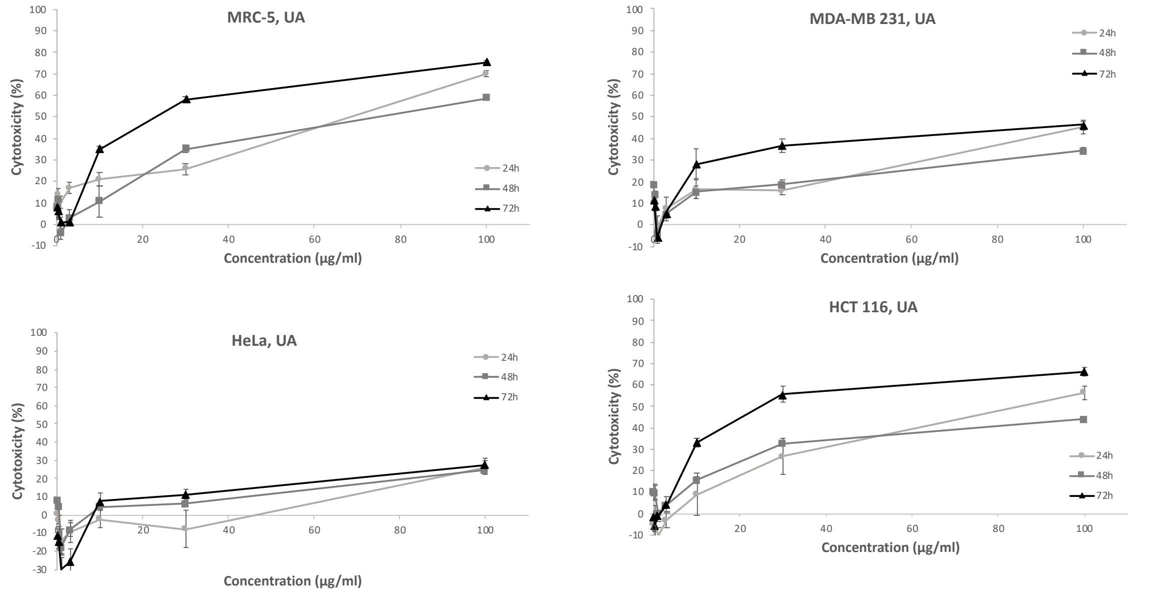

Figure S4. Dose-response curves of MTT assay after 24, 48 and 72h treatment of MRC-5, MDA-MB 231 HeLa and HCT 116 with usnic acid. The values are presented as mean ± SD of quadriplicates from at least three independent experiments.

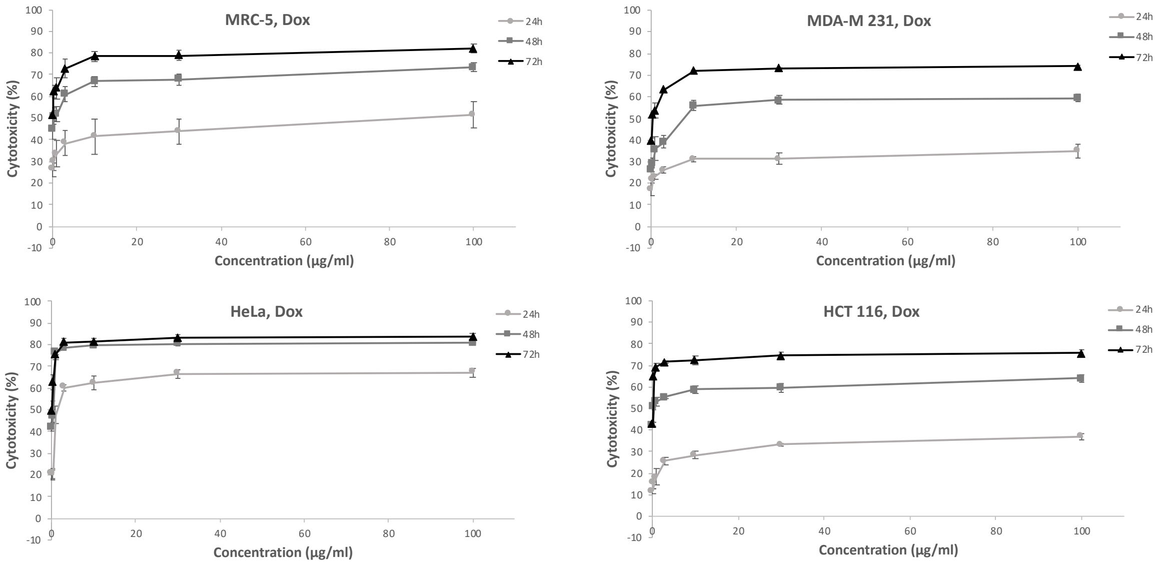

Figure S5. Dose-response curves of MTT assay after 24, 48 and 72h treatment of MRC-5, MDA-MB 231 HeLa and HCT 116 with doxorubicin. The values are presented as mean ± SD of quadriplicates from at least three independent experiments.
